# Hot Embossing to Fabricate Parylene-Based Microstructures and Its Impact on the Material Properties

**DOI:** 10.3390/polym16152218

**Published:** 2024-08-03

**Authors:** Florian Glauche, Franz Selbmann, Markus Guttmann, Marc Schneider, Stefan Hengsbach, Yvonne Joseph, Harald Kuhn

**Affiliations:** 1 Fraunhofer Institute for Electronic Nano Systems (ENAS), Technologie-Campus 3, 09126 Chemnitz, Germany; 2 Institute of Nanoscale and Biobased Materials (INBM), Technische Universität Bergakademie Freiberg, Gustav-Zeuner-Straße 3, 09599 Freiberg, Germany; 3 Institute of Microstructure Technology (IMT), Karlsruhe Institute of Technology, Hermann-von-Helmholtz-Platz 1, 76344 Eggenstein-Leopoldshafen, Germany; 4 Karlsruhe Nano and Micro Facility (KNMFi), Hermann-von-Helmholtz-Platz 1, 76344 Eggenstein-Leopoldshafen, Germany; 5 Center for Microtechnologies (ZfM), Technische Universität Chemnitz, Reichenhainer Straße 70, 09126 Chemnitz, Germany

**Keywords:** hot embossing, microstructures, nanostructures, Parylene, patterning, thermoforming, 3D structures, biocompatible, flexible substrate, XPS, XRD, FTIR, SEM

## Abstract

This study aims to establish and optimize a process for the fabrication of 3D microstructures of the biocompatible polymer Parylene C using hot embossing techniques. The different process parameters such as embossing temperature, embossing force, demolding temperature and speed, and the usage of a release agent were optimized, utilizing adhesive micropillars as a use case. To enhance compatibility with conventional semiconductor fabrication techniques, hot embossing of Parylene C was adapted from conventional stainless steel substrates to silicon chip platforms. Furthermore, this adaptation included an investigation of the effects of the hot embossing process on metal layers embedded in the Parylene C, ensuring compatibility with the ultra-thin Parylene printed circuit board (PCB) demonstrated previously. To evaluate the produced microstructures, a combination of characterization methods was employed, including light microscopy (LM) and scanning electron microscopy (SEM), X-ray diffraction (XRD), X-ray photoelectron spectroscopy (XPS), and Fourier-transform infrared spectroscopy (FTIR). These methods provided comprehensive insights into the morphological, chemical, and structural properties of the embossed Parylene C. Considering the improved results compared to existing patterning techniques for Parylene C like plasma etching or laser ablation, the developed hot embossing approach yields a superior structural integrity, characterized by increased feature resolution and enhanced sidewall smoothness. These advancements render the method particularly suitable for diverse applications, including but not limited to, sensor optical components, adhesive interfaces for medical wearables, and microfluidic systems.

## 1. Introduction

In recent years, the advancement in the creation of functional micro- and nanostructures has been substantial. This progress is largely attributed to the availability of adaptable materials, paving the way for tailored solutions across multiple applications. Central to this study is Parylene C, a thermoplastic polymer belonging to the poly(p-xylylene) family, exhibiting an array of excellent material properties. It offers optical transparency, low permeability to moisture and gas, minimal friction, and high thermal stability [1,2,3,4]. Additionally, its biostability and biocompatibility are confirmed according to ISO 10993 and FDA class VI standards [5,6]. These attributes collectively establish Parylene C as an optimal choice in conjunction with micro- and nanotechnologies, particularly as an adhesive for wafer and chip bonding, as well as a substrate for flexible electronics [7,8,9,10,11].

The structuring of Parylene C can be achieved by a variety of plasma-based methods. These include plasma etching, reactive ion etching (RIE), reactive ion beam etching (RIBE), high-density plasma etching (HDPE), and Bosch-like etching using switched chemistry [12,13,14,15]. For these energy-intensive processes, a masking step is required, elevating both time and financial costs. Moreover, these methods typically induce surface defects and lead to an enhanced roughness, which significantly degrades the side wall surface quality of the resulting structures. Such imperfections render etched micro- and nanostructures unsuitable for applications demanding high surface smoothness, like optical components. Alternatively, Parylene C can be patterned by laser ablation, which eliminates the need for additional mask materials. However, laser ablation is inherently a sequential process, making it a more time-intensive method [16].

A more promising approach for structuring Parylene C is hot embossing. This technique utilizes a heated mold insert, also called a shim, mostly fabricated in nickel, to imprint desired patterns on a substrate. Parylene’s inherent thermoplastic properties allow the direct application of hot embossing, bypassing any need for intermediate conditioning steps. This process particularly entails annealing Parylene C at temperatures surpassing its glass transition point (90 °C for Parylene C) and melting point (290 °C for Parylene C) in a vacuum, thereby molding it into the intended shape under physical constraints by the shim [17]. This method offers several benefits over established techniques: less process steps are required, and hence, the process is more cost-effective, as well as it promises the fabrication of high-precision micro- and nanostructures with an improved quality, which is essential for sensory and microfluidic applications for instance.

As shown in Table 1, hot embossing is a standard approach for patterning polymers for various uses, such as optical or biomedical applications. Hence, the common dimensions for hot-embossed structures found in the literature are around less than 10 µm to 100 µm [18,19,20,21,22,23].

**Figure 1:**
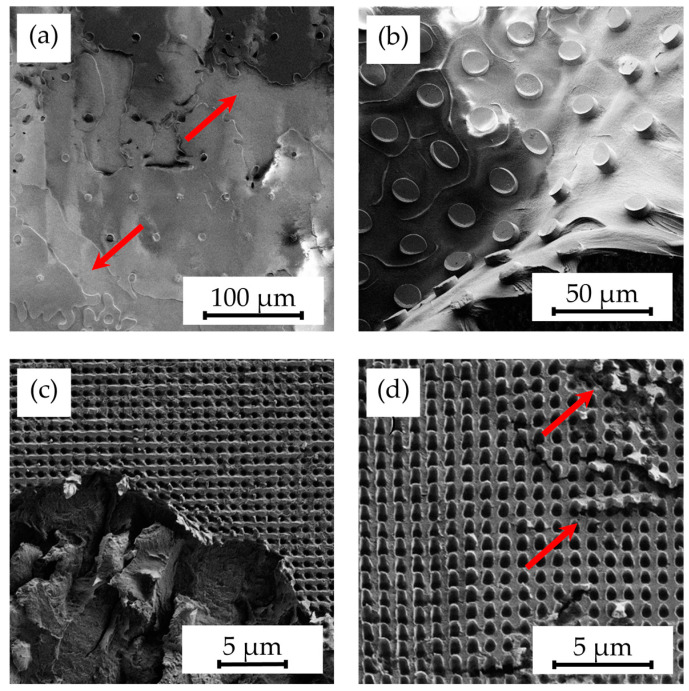
SEM images illustrating delamination scenarios during the hot embossing process, including (**a**) partial lack of patterning (indicated by arrows) due to detachment, (**b**) complete delamination of Parylene C layers, (**c**) tearing of layer fragments, and (**d**) cracks in Parylene C (indicated by arrows) on unannealed samples.

However, initial experiments using standard hot embossing techniques for patterning Parylene C encountered significant challenges. As illustrated in Figure 1, these included incomplete structuring (Figure 1a), detachment of the layer from the substrate (Figure 1b), adherence of Parylene C fragments to the mold insert (Figure 1c), and crack formation within the Parylene C layer (Figure 1d). These issues were primarily attributed to the low adhesion of Parylene C to its substrate, coupled with the tendency of molten Parylene C to stick to the mold insert. Addressing these challenges through the investigation and optimization of hot embossing parameters became a focal point of this research.

Incorporating hot embossing for the micro- and nanostructuring of Parylene C into standard semiconductor processes marks an important step for its broader application. A key focus was thus placed on employing this technique to create Parylene-based microstructures on silicon chips to enable further processing. Considering the use of Parylene C for encapsulation and as a material for ultra-thin, flexible printed circuit boards (PCBs), the impact of hot embossing on underlying structures is crucial. Due to the high forces applied to the layers, there is a risk of damaging these embedded levels. Ideally, the process should alter only the top Parylene C layer, leaving buried structures such as metallic redistribution layers (RDLs) intact.

This study entails the establishment of a reproducible hot embossing process and the optimization of the process parameters by investigating the impact of the process conditions on the material Parylene C. Furthermore, the feasibility of integrating the optimized hot embossing process at the chip level and its effects on embedded metallic layers were evaluated. Doing so, the novelty of the presented work is demonstrated not only in the fabrication of high-precision Parylene C microstructures with improved feature resolution and sidewall smoothness but also the comprehensive evaluation of the influence of hot embossing on the material properties.

## 2. Materials and Methods

### 2.1. Shim Fabrication

**Figure 2:**
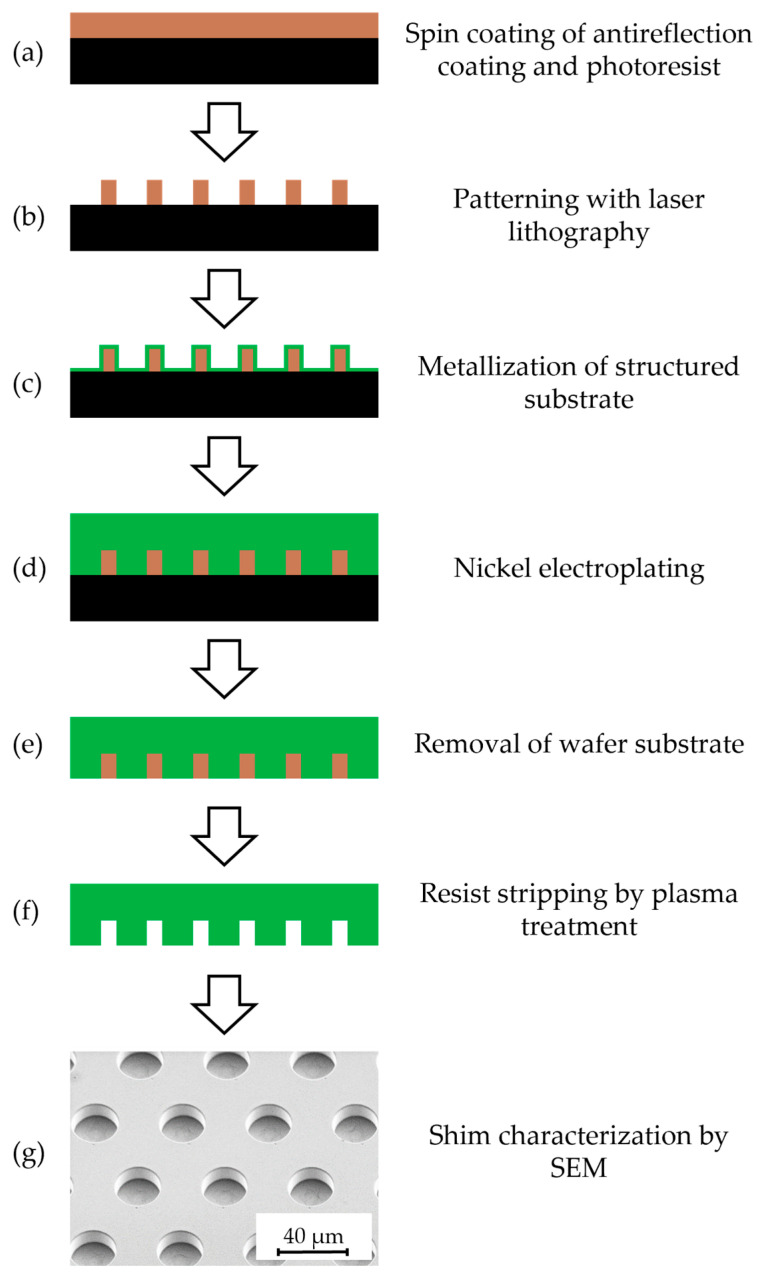
Process flow for the fabrication of the nickel shims featuring the inverted pattern.

To investigate the adhesive properties of different microstructures, a variety of pillar microstructures with varying diameters and heights were utilized on the shims. Specifically, microstructures with diameters of 10 µm and 20 µm, a pitch of 20 µm, 50 µm, and 80 µm between the pillars, and heights of 10 µm, 20 µm, and 30 µm were fabricated. Several nickel shims were realized according to the fabrication steps listed in Figure 2. The processes were performed on 4” silicon wafers as the master substrate. During pretreatment, these wafers underwent a cleaning stage using an oxygen plasma treatment at 100 W for 10 min, employing an RIE plasma etcher Etchlab 200 from SENTECH Instruments GmbH, Berlin, Germany.

(a) This was followed by the application of an AZ Barli II-200 antireflection layer (manufactured: Merck Performance Materials GmbH, Wiesbaden, Germany; filled: MicroChemicals GmbH, Ulm, Germany). The layer was spin coated at 3000 rpm for 30 s and subsequently tempered on a hotplate at 200 °C for 60 s. A similar process was applied for a 10 µm photoresist layer of mr-X 10 (micro resist technology GmbH, Berlin, Germany) by spin coating at 2000 rpm for 60 s and tempering at 95 °C for 10 min to remove the solvent.
(b) The next step involved patterning a 10.2 mm × 10.2 mm area of the photoresist layer with pillar structures with a diameter of 10 µm and 20 µm, as well as depths of 10 µm, 20 µm, and 30 µm. The pitch between the individual pillars was 20 µm, 50 µm and 80 µm for different shims, respectively. The patterning was achieved at a power setting of 185 mW using a maskless Laserwriter DWL66fs (Heidelberg Instruments Mikrotechnik GmbH, Heidelberg, Germany) equipped with a ND:Yag laser (λ = 365 nm) and by use of a 10 mm write head. After exposure, a post-exposure bake in the oven UT 5050 LAF (Kendro Laboratory Products GmbH, Langenselbold, Germany) was carried out. The oven was ramped up to 75 °C in 15 min and returned to RT in 4 h without an additional holding time. During this process, the photoresist crosslinked at the exposed areas. Afterwards, uncrosslinked photoresist was dissolved with a developer like q-methoxy-2-propanol acetate (PGMEA) and isopropanol for 12 min and 15 min, respectively.
(c) Prior to electroplating, a thin metallic seed layer consisting of 7 nm chromium and 45 nm to 50 nm gold was deposited onto the structured master substrate by the use of PVD. Doing so, the UNIVEX 450 vapor deposition system (Leybold GmbH, Cologne, Germany) was used, with chromium being coated at 5–6 Å/s and gold at 7–9 Å/s in a high-vacuum environment (≤10^−6^ mbar).
(d) Nickel electroforming followed, utilizing a boric acid containing nickel sulphamate electrolyte and current densities ranging from 0.1 A/dm^2^ to 1.5 A/dm^2^ to deposit an 800 µm to 1000 µm thick nickel layer on top of the master substrate.
(e) The removal of the master substrate was carried out either by mechanically lifting off the silicon wafer or by etching in KOH, in the cases where mechanical lifting posed a risk of structural deformation or was unfeasible due to geometrical constraints.
(f) Subsequently, the remaining antireflection and photoresist mask was also removed, requiring two etching steps: first with oxygen plasma at 100 W for 2 min using the RIE plasma etcher Etchlab 200 (SENTECH Instruments GmbH, Berlin, Germany), and then at 1200 W at 22 °C for 15 min using an R3T (Muegge GmbH, Reichelsheim (Odenwald), Germany).
(g) The structure quality of the shim was assessed using SEM.
(h) Finally, the remaining nickel block was then wire eroded to the desired height and final outer dimensions with a 200 µm brass wire at a tensile force of 900 J/mm^2^ using a Mitsubishi DWC 90 SZ (Mitsubishi Electric Corporation, Tokyo, Japan), before transferring it for use in the hot embossing process.

### 2.2. Sample Preparation

**Figure 3:**
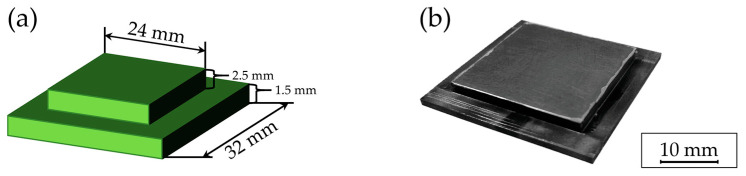
Schematic illustration (**a**) and photographic depiction (**b**) of the stainless steel samples.

For the experimental setup, two types of substrates were utilized: durable stainless steel substrates primarily employed for exploring variations in process parameters, and silicon chip stacks designed for hot embossing experiments at the chip level. The stainless steel substrates comprised a two-layered base plate, as shown in Figure 3, and were fabricated by wire eroding with a geometry specific for the hot embossing tool. For wire eroding, a Mitsubishi DWC 90 SZ eroding tool (Mitsubishi Electric Corporation, Tokyo, Japan) was used with a 200 µm brass wire at a tensile force of 900 J/mm^2^.

**Figure 4:**
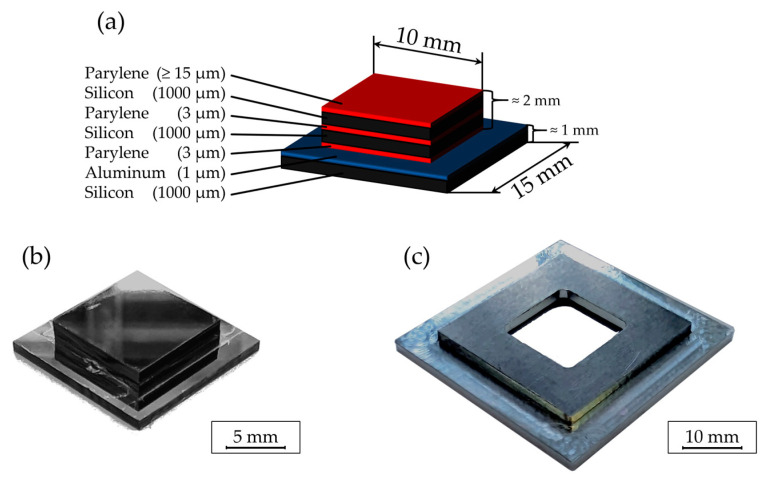
Schematic illustration (**a**) and photographic depiction (**b**) of the chip stack assembly, accompanied by (**c**) its respective holder designed for the hot embossing system.

The silicon sample design is illustrated in Figure 4a,b. A chip stack design was implemented, consisting of a 1 mm thick silicon chip coated with a thin aluminum layer at the base, facilitating the subsequent separation of chips using aluminum wet etching in KOH. Two additional 1 mm thick silicon chips coated with a 3 µm Parylene C layer were bonded to this base chip using a T-3002-FC3 chip bonder (Dr. TRESKY AG, Thalwil, Switzerland). Bonding parameters included a temperature of 300 °C, a duration of 30 s, and an applied force of 6.45 N [11].

Furthermore, a special holder (Figure 4c) compatible with the embossing equipment was developed to securely position the chip stack. Once mounted, the holder provided additional stability and prevented dislodgement as well as adhesion to the mold.

**Figure 5:**
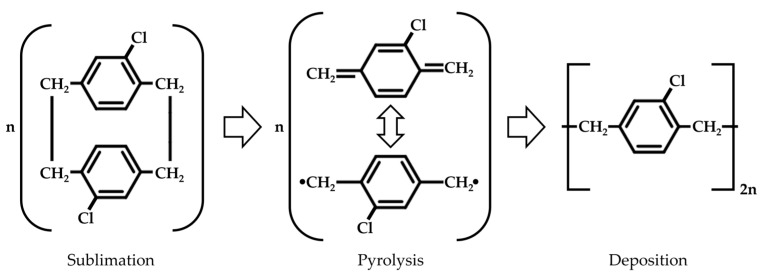
Molecular composition of the three-step CVD process of Parylene C according to Gorham [24].

All Parylene C coatings were applied using the Plasma Parylene LC 300 RW system from Plasma Parylene Systems GmbH, Rosenheim, Germany. Doing so, the chemical vapor deposition (CVD) of Parylene C according to Gorham [24] was reproduced, which consists of the three steps depicted in Figure 5. Initially, the commercially available, powdery dimer undergoes sublimation at 130 °C. In the pyrolysis zone, the gaseous dimer is exposed to a temperature of 740 °C, leading to the thermal cracking of the dimer into monomers. Finally, by cooling the monomers down to ambient temperature, linear polymer chains are formed through a condensation-like polymerization reaction, thereby preventing intrinsic stress within the Parylene C layers [24]. This entire procedure is executed under vacuum conditions. Subsequently, a thickness reflectometer F20 from Filmetrics GmbH, Unterhaching, Germany, was used to measure the resulting layer thickness.

For bonding of the substrate layers, only 3 µm Parylene was deposited, while for the embossing, a 10 µm to 30 µm thick layer of Parylene C was used. Prior to the Parylene C deposition, the substrates underwent various surface treatments to tune substrate adhesion. These treatments included milling, sanding with P60 grit sandpaper, and sandblasting using both glass beads and granules. Some substrates received a combination of these methods. The impact of these treatments on the adhesion between the substrate and Parylene C was investigated and compared to the adhesion on unprocessed substrates. With the intention of improving adhesion between the substrate and Parylene C, some 15 µm thick Parylene C samples underwent a heat treatment at 300 °C for 30 min in a vacuum using an EVG-540 bonder from EV Group Europe & Asia/Pacific GmbH, Sankt Florian am Inn, Austria, after the Parylene C deposition. To investigate the effects of hot embossing on the layers embedded in Parylene C, the chip assembly was enhanced with eight additional layers to mimic the architecture of actual multilayer chip configurations: four gold layers, each 300 nm thick, to simulate buried metallic redistribution layers, were alternated with four additional Parylene C layers, each 3 µm thick. The gold layers were deposited using physical vapor deposition (PVD).

### 2.3. Hot Embossing Process

**Figure 6:**
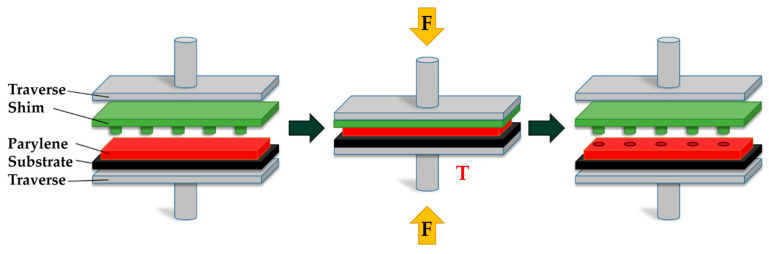
Hot embossing process of Parylene C used for processing the samples in this work with F representing the applied force and T the increased temperature.

The general process sequence of the hot embossing is shown in Figure 6. The system utilized for this process is a modified tensile testing machine, specifically a ZwickRoell GmbH & Co. KG (Ulm, Germany) model, integrated with a control unit from JENOPTIK AG, Jena, Germany. These modifications were carried out by the Institute for Microstructure Technology (IMT) at the Karlsruhe Institute of Technology (KIT).

In the initial phase of the process, both the Parylene-coated substrate and the shim were mounted onto the traverses accordingly. Following the initial setup, the process chamber was sealed and evacuated. Subsequently, by heating the traverses, the Parylene C was brought to a molten state at temperatures exceeding its melting point. During this phase, the shim mounted on the traverse was pressed into the molten Parylene C to imprint the desired structures.

After the heating phase, the system was allowed to cool down under vacuum conditions. The opening of the machine was then executed at a controlled rate, allowing for the vertical demolding of the Parylene C structures. To enhance the detachment of the shim from the Parylene C, a silicon-free and biocompatible, NSF-registered Lusin Alro OL 202 F release agent (Chem-Trend GmbH, Norderstedt, Germany) was applied to the Parylene C layer before positioning. Despite efforts to achieve reproducibility by applying a consistent amount of release agent, manual spray coating resulted in a variability in the deposited amount. An optimized method involved a uniform coating of the sample surface with the release agent for 1 s from a distance of 30 cm. Finally, the samples and shims were removed from their respective traverses and frames. For reuse, all shims underwent a cleaning process using oxygen plasma at 500 W for 90 min in a microwave plasma tool Q245S by Plasma Parylene Systems GmbH, Rosenheim, Germany.

To investigate the parameters influencing the hot embossing process of Parylene C, a series of experiments were conducted, focusing on variations in embossing temperature and force as well as demolding speed and temperature on 15 µm thick Parylene C layers.

The hot embossing temperature was varied between 295 °C, 305 °C, 315 °C, 325 °C, and 350 °C, with the initial temperatures slightly exceeding and the higher temperatures substantially surpassing the melting temperature of Parylene C [9]. Furthermore, the hot embossing force was varied among 15 kN, 20 kN, and 25 kN—the latter representing the maximum force achievable with the embossing equipment. Adjustments to the demolding speed included settings of 1.0 mm/min, 0.5 mm/min, and 0.2 mm/min. Lastly, the demolding temperature was investigated at 100 °C, 60 °C, 40 °C, and a near-room temperature at 30 °C, optimizing for process time. For the parameters not being varied, experiments were conducted under constant conditions: a hot embossing temperature of 305 °C, a hot embossing force of 25 kN, a demolding speed of 0.2 mm/min, a demolding temperature of 40 °C, and a Parylene C layer thickness of 15 µm, employing a shim to create 20 µm wide and 10 µm high pillar structures with a 50 µm pitch.

In investigating the dimensional limits and quality achievable through the hot embossing of Parylene C, layer thicknesses were varied from 10 µm to 30 µm to accommodate different structural dimensions. Typically, the semiconductor industry employs layer thicknesses up to 15 µm, yet for the creation of higher structures nearly identical in height, layers of 30 µm were utilized.

### 2.4. Material and Structure Characterization Methods

XRD, XPS, and FTIR were utilized to characterize the samples. The Bruker D8 Advance instrument (Bruker AXS GmbH, Karlsruhe, Germany) was employed for the XRD measurements. The device operated using Cu-Kα radiation at 40 kV and 40 mA, set in a parallel beam geometry. This setup included a nickel/carbon multilayer mirror, a secondary 0.12 ° soller collimator, a secondary lithium fluoride monochromator, and a semiconductor scintillation counter.

The XPS analysis was performed using the ESCALAB 250Xi (Thermo Fisher Scientific, Waltham, MA, USA). Measurements were conducted with spot diameters of 250 µm and 300 µm, pass energy of 100 eV, and a resolution of 0.25 eV. Unwanted charging of the Parylene C layer was negated by applying a small opening through the layer near the analyzed area. The resulting data were subsequently analyzed using CasaXPS software (ver. 2.3.25PR1.0) with the application of a linear background correction.

For the FTIR measurements, the VERTEX 70 FTIR spectrometer (Bruker Corporation, Billerica, MA, USA) in the range of 3300 cm^−1^ to 800 cm^−1^ was used. The FTIR measurements were conducted via transmission through 10 µm thick Parylene C membranes. Additionally, the FTIR spectrum for the background was independently measured and subsequently deducted from each sample’s spectra. To rectify the sinusoidal distortions observed in the FTIR spectra, a manual sinus function fitting was applied to the raw data. This process involved adjusting the signal intensity, I [a.u.], as a function of the wave number, k [cm^−1^], utilizing the sinus function’s parameters: amplitude A [a.u.], wavelength B [cm], phase shift C [a.u.], and vertical shift D [a.u.], as given in Equation (1). The correction entailed subtracting the fitted sinus function from each spectrum [8].

(1) $$I\left(k\right)=I\cdot \left(\frac{1}{\lambda }\right)=A\cdot \sin\;\left(B\cdot k+C\right)+D$$

The surface topography of the hot-embossed structures was evaluated using a DektakXT profilometer from Bruker Corporation, Billerica, MA, USA, and detailed surface imaging was obtained through confocal microscopy with a Nikon CL 200 microscope by Nikon Corporation, Tokyo, Japan. Additionally, top-view imaging of the structures utilized two methods: light microscopy (LM) via an Eclipse L200 microscope (Nikon Corporation, Tokyo, Japan) and SEM with a GAIA3 FIB-SEM by TESCAN GmbH, Dortmund, Germany. For cross-sectional analysis, the samples were embedded in epoxy resin and examined using SEM.

## 3. Results and Discussion

### 3.1. Optimization of the Hot Embossing Shim Quality

The initial fabrication of nickel shims revealed a suboptimal adhesion of the electrodeposited nickel on the metallized lithographically prepared master substrates, leading to plating defects and detachment of the nickel layer. An improvement was observed when 2000 µm thick silicon substrates were used instead of the thinner 500 µm to 1000 µm ones, which provided a more stable base for electroplating, resulting in structures with satisfactory adhesion.

**Figure 7:**
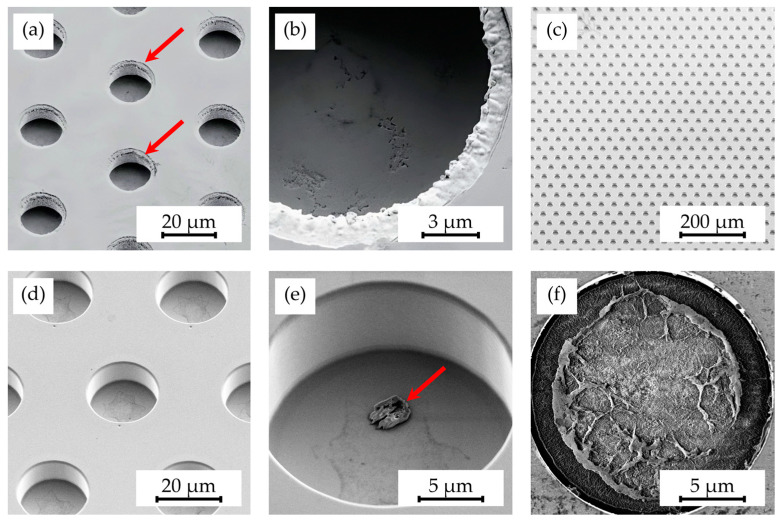
SEM images depicting microstructures on a shim after electroplating, showing (**a**) rough sidewalls (indicated by arrows) in the cavities and (**b**) unintended material undercuts, alongside (**c**) a fully processed shim with (**d**) defect-free structures, as well as shims featuring (**e**) minor resist residues (indicated by arrows) and (**f**) extensive resist residues.

However, inconsistencies remained post-electroplating, notably the quality of the sidewalls in inverted structures, characterized by roughness and undercuts caused by undesired nickel growth, as depicted in Figure 7a,b. These imperfections directly impacted the hot embossing process of Parylene C layers, causing difficulties in demolding due to entrapment in cavities and increased friction from rough sidewalls, leading to the partial or complete delamination of Parylene C layers.

Efforts to resolve these issues included the iterative adjustments of various process parameters, such as laser-writing power. Despite these attempts, consistent results were elusive. Several factors were hypothesized to influence these outcomes: the thicker silicon substrate may have altered the lithography process due to less warping during processing and thus better reflective properties and the laser-writing parameters, especially power, could be crucial, and environmental factors like ambient temperature might significantly affect electroplating.

Nevertheless, subsequent fabrications yielded better results with defect-free structures, depicted in Figure 7c,d, though occasional photoresist residues in cavities, shown in Figure 7e,f, required removal with a plasma etcher or ultrasonic acetone baths.

### 3.2. Surface Treatments and Release-Agent Effects on the Delamination of Parylene C and Its Material Properties during Hot Embossing

In this study, various strategies to mitigate the delamination of Parylene C from substrates during hot embossing were explored. Initially, surface treatments including milling and abrasion were tested, under the assumption that an increased roughness might improve adhesion. Contrary to expectations, these methods did not enhance the adherence of Parylene C, indicating that substrate roughness does not play a significant role in this context.

**Figure 8:**
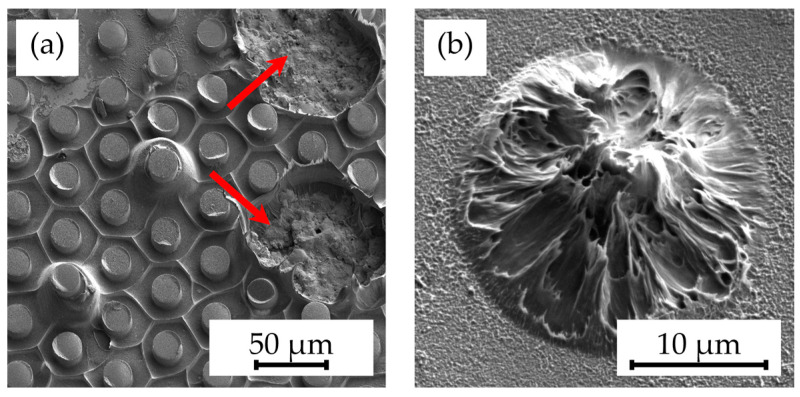
SEM images showing the detachments of (**a**) multiple pillars (indicated by arrows) and (**b**) individual pillar structures on samples annealed at 300 °C for 30 min caused by the hot embossing process at a 305 °C embossing temperature and 25 kN embossing force, as well as demolding at 40 °C with 0.2 mm/min.

Subsequently, the potential of annealing Parylene-coated samples before embossing, aiming to enhance the fusion between Parylene C and the substrate, was investigated. However, this approach failed to prevent delamination during hot embossing, as depicted in Figure 8.

To address these challenges, the application of a release agent during hot embossing was tested. This method successfully reduced friction and, consequently, the adhesion between the Parylene C layer and the shim surface, thereby diminishing delamination risks. Nonetheless, delamination still occurred at substrate edges where the release agent was more easily pressed out, leading to direct contact between Parylene C and the shim. This contact caused the sticking and partial tearing of the Parylene C, affecting the quality of the patterned structures and necessitating additional cleaning steps for the shims and the removal of release-agent residues from the microstructures.

**Figure 9:**
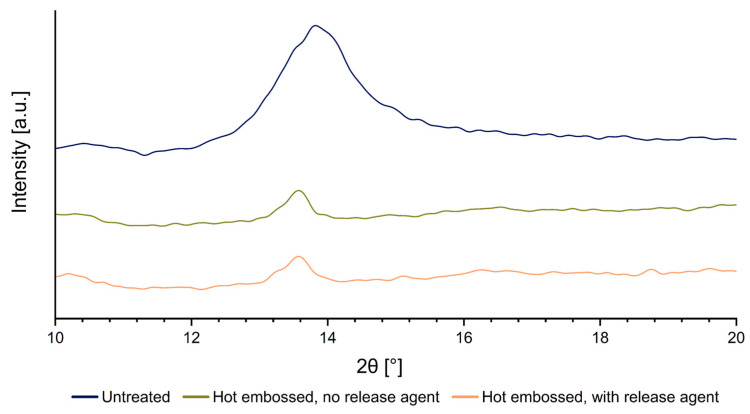
XRD measurements of a freshly coated Parylene C sample (blue), as well as samples without a release agent (green) and with a release agent (orange), both subjected to hot embossing.

To investigate the structural changes in Parylene C layers pre and post hot embossing, 15 µm thick Parylene C samples, both with and without a release agent, were subjected to a hot embossing process. This procedure involved a hot embossing temperature of 305 °C, a hot embossing force of 25 kN, and demolding conditions of a 40 °C cooling temperature and a 0.2 mm/min opening speed. In investigating the impact of release agents on the hot-embossed Parylene C material, this study utilized XRD, XPS, and FTIR analyses to determine changes in both crystalline structure and chemical composition. The XRD (Figure 9) analysis compared freshly Parylene-coated samples with those hot-embossed ones at 350 °C, with and without the release agent. Conducted over a 2θ range of 5 ° to 40 °, the analysis aimed to determine the impact on the crystalline structure of the Parylene C. Untreated samples exhibited characteristic peaks at 2θ~14 °, while embossed samples showed a shift to 2θ~13.7 °, indicating a modification in the crystalline structure, yet not a complete transition to an amorphous state [11]. Notably, the diffractograms of hot-embossed samples with or without the release agent were similar, suggesting the agent had no significant effect on crystallinity compared to the impact of the increased temperatures.

Additionally, the peak height and width of tempered samples decreased, signaling a reduction in Parylene C crystallinity, correlating with the potential brittleness upon exposure to high temperatures, which could impair the material’s properties.

**Figure 10:**
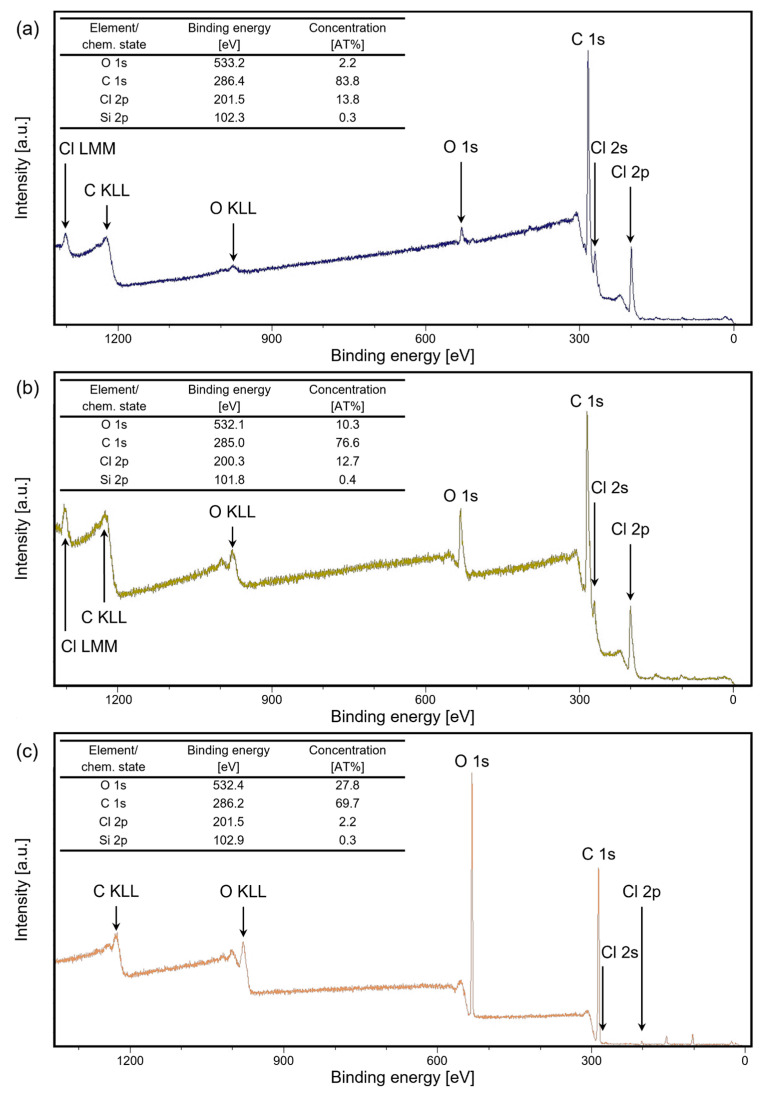
XPS measurements of (**a**) an untreated Parylene-coated sample, (**b**) a hot-embossed sample without the release agent, and (**c**) a hot-embossed sample with the release agent.

The XPS measurements depicted in Figure 10a–c provided insights into the chemical surface composition of Parylene-coated silicon chips post-embossing, with and without using a release agent. Compared to the untreated samples with only traces of oxygen from the sample preparation, a significant increase in oxygen content was observed, indicating a pronounced influence of surface modifications on the material’s chemical composition. Samples without a release agent exhibited a five-fold increase in oxygen content, suggesting the oxidation of Parylene C. This phenomenon implies that the vacuum conditions of around 10 mbar during the hot embossing process were insufficient to prevent oxidation, as demonstrated by another study where higher vacuum levels (<10^−3^ mbar) successfully mitigated such oxidation effects [8]. Given that the biocompatibility of oxidized Parylene C has not been conclusively proven, an additional motivation for transferring the hot embossing process to the chip or wafer level is, for instance, to utilize chip and wafer bonding equipment for hot embossing processes. This approach enables the execution of hot embossing processes under higher vacuum conditions, thereby potentially enhancing the overall process reliability and outcomes.

Conversely, samples coated with the release agent showed an even more substantial 13-fold increase in oxygen content. This result indicates that the use of the release agent negatively impacted the oxidation resistance of Parylene C, facilitating further oxygen penetration and subsequent oxidation. The analysis suggests two possibilities: either the Parylene C has undergone actual oxidation or residues of the release agent are present on the samples. While the chlorine content appeared reduced in samples with the release agent, this could be due to a relative shift in the proportions of carbon and oxygen rather than a true decrease in chlorine. The increased oxygen content might also be attributed to these residues, implying that an additional cleaning step after hot embossing might be necessary to remove any remaining release agent.

**Figure 11:**
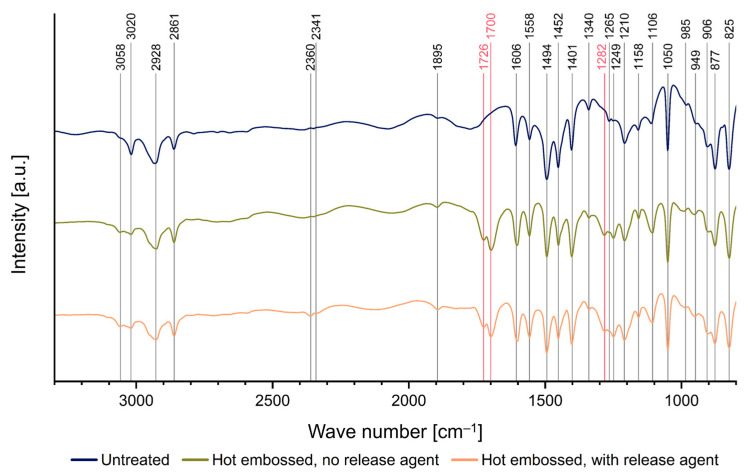
FTIR measurements of a freshly coated Parylene C sample (blue), a sample without the release agent (green), and a release-agent-treated sample (orange), both hot embossed at 305 °C; the positions of the carbonyl bond peaks at 1282 cm^−1^, 1700 cm^−1^, and 1726 cm^−1^ are highlighted in red.

In addition to the XPS measurements, FTIR spectra were recorded to obtain depth signals and insights into the influence of process parameters within the bulk material, as illustrated in Figure 11. The corresponding modes for each wave number are listed in Table 2. The hot-embossed samples show peaks at 1282 cm^−1^, 1700 cm^−1^, and 1726 cm^−1^ signifying carbonyl bonds, which are indicators of Parylene C oxidation that are lacking in the untreated sample [25,26,27,28].

The results indicate that the hot embossing process induces oxidation reactions not only on the surface but also within the bulk of the Parylene C layer, altering its chemical composition. Furthermore, the presence of carbonyl bonds indicative of oxidation was comparable in the Parylene C layers of embossed samples, regardless of release-agent application. Consequently, employing a release agent prior to embossing is preferable for better demolding, despite the potential alterations in surface composition as evidenced by the XPS analysis. Furthermore, due to the excellent barrier properties of Parylene C, a chemically pristine surface can be easily restored by applying an additional coating of Parylene C of already less than 500 nm without changing the structure dimensions significantly.

In conclusion, despite process refinements and different approaches, preventing Parylene C delamination during hot embossing without employing a release agent remains a significant challenge.

The application of a release agent introduces negative effects such as increased oxidation and potential residue contamination, which require additional cleaning steps. Future work should focus on optimizing hot embossing conditions, exploring alternative release agents, or developing new surface treatments to enhance adhesion without compromising the integrity of the Parylene C layer.

### 3.3. Optimization of the Process Parameters to Establish a Reproducible Hot Embossing Process

**Figure 12:**
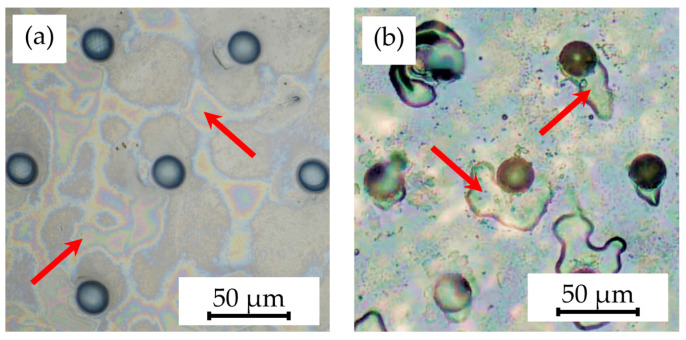
Light microscopy images of samples hot embossed at 305 °C with 25 kN of force and a demolding at 40 °C with 0.2 mm/min, showing (**a**) striation patterns (indicated by arrows) due to the release agent and (**b**) accumulations of residual release agent (indicated by arrows) near pillar structures.

In optimizing the process parameters for establishing a reproducible hot embossing process for Parylene-based microstructures, the investigation revealed several key findings. An overview of the results is given in Table 3. In the evaluation of structure quality across the tested hot embossing process temperatures, no significant discrepancies were observed. Similarly, no substantial differences were detected between stainless steel and silicon chip substrates within the examined temperature range, affirming the successful patterning across all tested temperatures. However, higher temperatures proved more efficient at removing the release agent, evident by the reduced residues post-embossing. Samples embossed at 295 °C and 305 °C exhibited increased streaks and accumulations of the release agent near structures, as seen in Figure 12a,b, which were not evident or not as prevalent at process temperatures of 315 °C and higher. This phenomenon can be attributed to the fact that Parylene C at higher temperatures such as 315 °C is either more fully melted or exhibits a lower viscosity, thereby facilitating a more uniform distribution and reducing the occurrence of release-agent accumulations. Thus, the choice between increasing temperature and post-process cleaning requires careful consideration, as lower temperatures are less aggressive on Parylene C, whereas temperatures above 315 °C could result in a higher degree of crystallinity in Parylene C, causing brittleness and lowering its mechanical stability [11,31].

**Figure 13:**
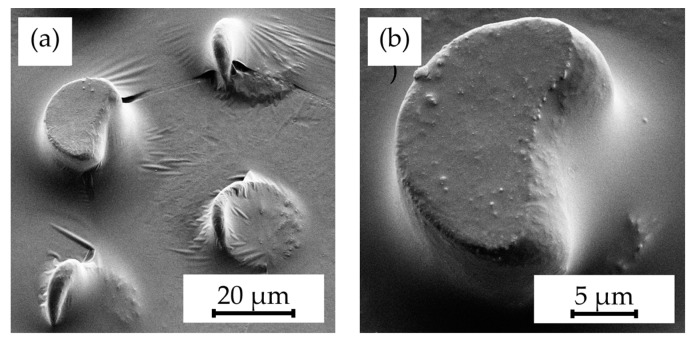
SEM images of (**a**) blurred and (**b**) incomplete structures resulting from release-agent flow into the shim after hot embossing with a force of 15 kN at 305 °C and demolding at 40 °C with a 0.2 mm/min opening speed.

A correlation was identified between the embossing force applied and the replication fidelity of microstructures, revealing that forces of 15 kN and 20 kN resulted in structural defects and an increased surface roughness, whereas a force of 25 kN significantly enhanced the precision of the inverted pillar structures on Parylene C, yielding smoother structures and a reduced roughness. The choice of surface roughness is dictated by the intended application, with a higher roughness potentially benefiting adhesion applications, while smoother surfaces are crucial for applications like optical waveguides to minimize light diffusion and maintain transmission efficiency. Furthermore, the application of 20 kN and 25 kN forces effectively displaced the release agent, preventing void formation and the accumulation of the agent, which at 15 kN was responsible for blurred structural edges and incomplete pillar formations, as depicted in Figure 13a,b. Despite these observations, the displacement of the release agent showed no significant difference between the forces of 20 kN and 25 kN.

At the chip level, the hot embossing process proved successful across the investigated forces. However, approximately 30 % of experiments encountered chip breaking, regardless of the force applied. This outcome contradicts the hypothesis that higher forces would increase chip breakage rates. The primary causes of chip breaking were identified as particles on the substrate creating pressure points and an uneven application of the mold due to equipment tolerance issues. These challenges, particularly the latter, are specific to the equipment used and are not easily negated.

The demolding speed and cooling temperature were found to significantly impact the delamination of Parylene C layers. A demolding speed of 1.0 mm/min or 0.5 mm/min resulted in a greater delamination on both stainless steel and silicon chip substrates compared to a slower speed of 0.2 mm/min. This increased delamination at higher speeds can be attributed to higher frictional forces. The friction decreased until the separation of the shim and sample, then increased due to a release-agent film, indicating a preference for a slower demolding speed of 0.2 mm/min for reduced delamination.

Regarding the demolding temperature, cooling the samples to just 100 °C or 60 °C resulted in the partial stripping of single-pillar structures or larger Parylene C spots, suggesting an excessive volumetric expansion at this temperature. Lower temperatures of 40 °C and 30 °C significantly reduced delamination by minimizing the volumetric expansion of the pillars, allowing for a more efficient demolding of microstructures compared to the higher temperature of 100 °C or 60 °C. Therefore, lower temperatures are recommended for demolding, despite the longer cooling time required, with no noticeable difference between demolding at 30 °C and 40 °C.

In this study, an optimal parameter set for the hot embossing trials was determined, as listed in Table 4. This parameter set was used as a baseline for subsequent experiments.

### 3.4. Investigation of Structure Dimension Limits and Quality Possible by the Hot Embossing of Parylene C

**Figure 14:**
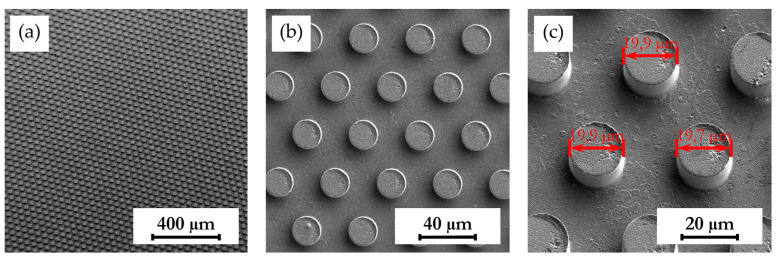
SEM images depicting 20 µm high and 20 µm wide pillars with a 20 µm pitch (**a**,**b**) in a large uniformly patterned area of Parylene C with different magnifications and (**c**) with dimension annotations; all shown structures are 20 µm high and were fabricated by hot embossing at 305 °C with 25 kN of force and demolding at 40 °C with 0.2 mm/min on a 15 µm thick Parylene C layer.

In exploring the dimensional constraints and fidelity achievable through the hot embossing process of Parylene C, shims with various microstructures were evaluated, as evidenced in Table 5. The process proved capable of fabricating features as small as 10 µm in diameter and height. However, these structures exhibited an increased tendency for individual pillars to detach from the patterned area, suggesting a propensity for easier separation attributable to their minimal dimensions. Attempts to replicate the structures of even smaller dimensions were unsuccessful, underscoring the necessity for additional research in this area. In terms of structure height, it was possible to hot emboss micropillars on Parylene C with diameters of 20 µm and heights of 10 µm, 20 µm, and 30 µm (Table 5). This resulted in achievable aspect ratios of up to 1.5 using the parameters selected for this study. Additionally, the surface roughness of the structures was notably low (Table 5). This suggests that the demolding occurred homogeneously, with minimal friction effect and without significant adhesion within the mold cavities, a factor that is crucial for the integrity and functionality of the structures. The dimensions of the microstructures match those of the inverse structures of the mold, with negligible deviations of merely 0.1 µm to 0.5 µm in diameter, as depicted in Figure 14c, on structures fabricated using a shim with a 20 µm width and 10 µm high pillars. Structures with a 10 µm diameter showed deviations in the same range, indicating that there is little to no volumetric shrinkage during the hot embossing and subsequent cooling phases. This implies that structures of this scale can be precisely transferred onto Parylene C. SEM images illustrated the pillar structures with high uniformity and minimal defects across the Parylene C substrate, as shown in Figure 14a,b. This homogeneity suggests the high quality of the shim used and the optimization of the process parameters employed in fabrication.

**Figure 15:**
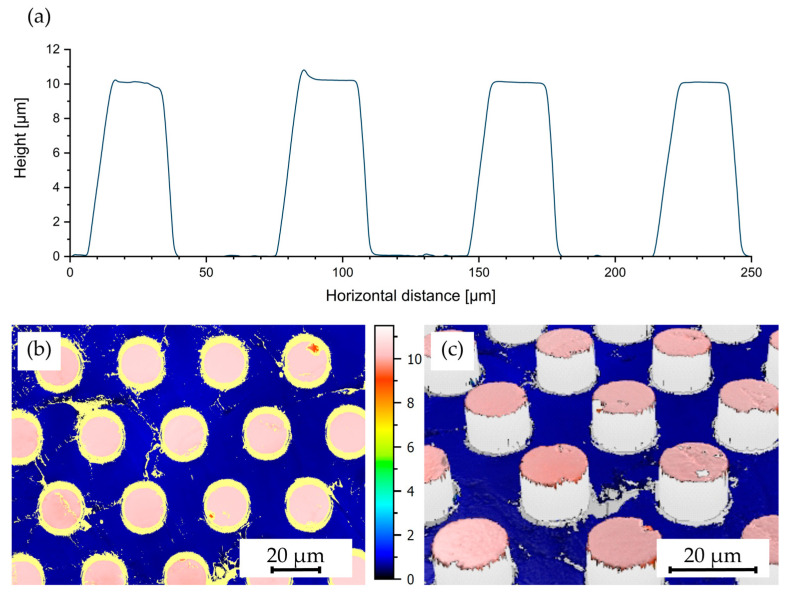
(**a**) Profilometer measurements of a sample produced by hot embossing at 305 °C with 25 kN of force and demolding at 40 °C with 0.2 mm/min featuring pillars with dimensions of 10 µm in height, 20 µm in width, and 50 µm in spacing, as well as (**b**,**c**) confocal microscopy measurements of a sample with pillars measuring 10 µm in height, 20 µm in width, and 20 µm in spacing; (**b**) in top view and (**c**) at an inclined angle.

Further analysis on the structure height was conducted, with results presented in Figure 15a, displaying data from the profilometer measurements of structures aimed to be 10 µm in height. The measured heights met the target structure height, verifying again the absence of significant volumetric shrinkage. The noticeable slope on the second pillar is explained by delamination effects, such as a weak partial adhesion to the shim due to irregularities. Confocal measurements, as illustrated in Figure 15b,c, confirmed the homogeneity of the structure heights across the patterned area. The measurements also highlighted the well-defined edges that could be achieved. However, it was observed that the pillar structures in some instances are exhibiting a consistent slope on the top side rather than being perfectly planar. Measurements confirmed a height of 2 µm to 3 µm for these steps. The uniformity of this feature across all pillars suggests a structural origin, possibly due to a slight tilt during the embossing process caused by the play in the mold holder, leading to the angled surface upon demolding. Nevertheless, the potential impact of this variation must be carefully evaluated for subsequent structuring processes.

### 3.5. Impact of Hot Embossing on Silicon Chips with Buried Metal Layers

**Figure 16:**
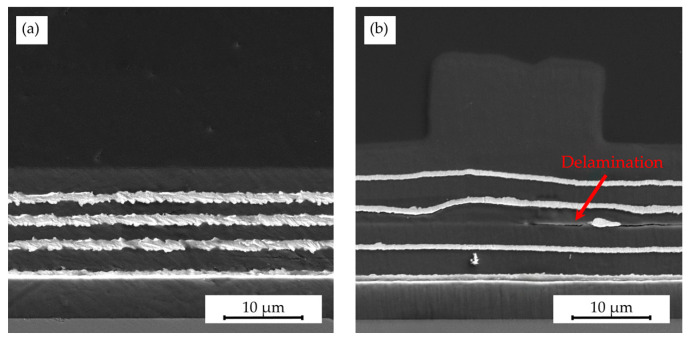
SEM images of the silicon chip with eight layers alternating between gold (light gray, 300 nm) and Parylene C (dark gray, 3 µm) (**a**) before hot embossing with rough gold layer edges resulting from the sample preparation and (**b**) after the hot embossing process presenting a delaminated area with a gold particle most likely introduced during the sample preparation; the embossing process employed a hot embossing temperature of 305 °C, force of 25 kN, demolding speed of 0.2 mm/min, and a demolding temperature of 40 °C, utilizing a shim featuring 10 µm high structures, 20 µm in diameter, and spaced 50 µm apart.

To explore the impact of hot embossing on embedded metal layers, experiments were conducted with gold layers encapsulated within Parylene C. SEM images of cross-sections from the gold–Parylene C stacks before and after embossing (Figure 16) provide a clear indication of the influence of the structuring process. Notably, the Parylene C layers seemed stretched post-embossing, thereby increasing in height, with these effects being more pronounced in the upper layers, indicating a depth-dependent influence. In contrast, the difference in metal layer thickness, as well as roughness, before and after hot embossing can be attributed to the preparation of the SEM samples and was not influenced by the hot embossing process itself.

Additionally, the delaminated area observed in the third Parylene C layer likely formed during demolding, suggesting that multilayered Parylene C systems are at risk of delamination when structured by hot embossing. A gold particle found within the delaminated area was likely introduced during the cross-sectional sample preparation, such as dicing or polishing, given the improbable particle integration during Parylene C deposition using CVD under vacuum conditions.

In conclusion, the structuring process is conditionally suitable for multilayer systems if the changes in layer thickness, i.e., expansion of Parylene C layers, are acceptable for subsequent processing and application. Care must be taken to ensure an adequate adhesion within and between layers to prevent delamination. Furthermore, if the occurrence of delamination is a depth-dependent phenomenon, it might be mitigated by increasing the thickness of the topmost Parylene C layer, as well as placing functional layers in the lower 50 % to 60 % of the total stack thickness. However, additional systematic experiments are necessary to substantiate this approach.

## 4. Conclusions

In conclusion, this study established a reproducible hot embossing process for the fabrication of 3D structures in Parylene C and, hence, demonstrates a significant step forward in the realization of Parylene C microstructures. The optimization of process parameters was a key development, achieving more consistent and high-quality microstructures. The integration of hot embossing into chip-level patterning also showed promising results, further demonstrating the technique’s applicability in semiconductor processes and potential for the microstructuring of other materials as well. Overall, this study provides a foundational understanding of hot embossing for Parylene C microstructures and sets a direction for future advancements in this area. Looking forward, this study opens up new ways for the application of Parylene C in new fields. The potential for creating optical elements, small-scale energy generation devices, ultra-thin circuit boards, and microfluidic systems is particularly promising. Future research should focus on developing a release-agent-free solution and less intrusive embossing parameters, particularly by lowering temperatures and improving the vacuum conditions, to minimize the undesired surface effects and prevent internal oxidation, as demonstrated in other studies [8]. Thereby, the integrity and functionality of the Parylene-based micro- and nanostructures could be increased. Furthermore, a research focus should be put on transferring the process to larger substrates by adapting and refining especially the process parameters on a wafer-level basis, as well as exploring the creation of smaller as well as reproducible hierarchical structures.

## Figures and Tables

**Table 1:** State-of-the-art overview of materials, structures, and applications achieved through hot embossing.

Hot-Embossed Material	Hot Embossing Parameters	Realized Structures	Application	Reference
Chalcogenide glass of 1.5 mm thickness	0.4–0.8 MPa 250 °C	Diameter of 20 µm and pitch of 30 µm	Microlens arrays	[18]
PMMA sheet of 1mm thickness	20 kN 180 °C	Depth of 27 µm and width of 110 µm	Microchannels for biomedical applications	[19]
Polycarbonate of 0.3 mm thickness	17.2 MPa 180–200 °C	Dimensions of >100 µm	Micromirrors for micro-optical switches	[20]
PMMA sheet of 1 mm thickness	20 kN 180 °C	Depth of 30 µm, width of 50 µm, and pitch of 50 µm	Microfeature slots for DNA/RNA tests	[21]
Thermal curable polymer (ZP2145M)	10 MPa 250–280 °C	Area of 6.8 × 7.5 µm^2^	Optical waveguides	[22]
Parylene C of 60 µm thickness	2 kN 270 °C	Depth of 25 µm, length of 1 mm, width of 10 µm, and pitch of 10 µm	Micromold replication	[23]

**Table 2:** FTIR vibrational nodes in reference to the designated wave number; the table is based on [25].

Wave Number k [cm^−1^]	Mode	Reference
826	H–C–H two neighboring hydrogen atoms bonded to the ring, C–C aliphatic stretching, C–H out-of-plane wagging	[27,28,29,30]
878, 906, 985	C–H single hydrogen to the ring, out-of-plane wagging	[28,29,30]
1051	C–H in-plane bending, C–Cl bonding to the ring	[28]
1110, 1265	CH_2_ wagging	[29,30]
1282	Parylene C Oxidation (C=O stretching)	[30]
1340, 1401	C=C semicircle stretching	[29,30]
1452	CH2 on the benzene ring, CH_2_ deformation	[27]
1494	C–C aromatic stretching	[27]
1558, 1606	C=C ring stretching	[29,30]
1700, 1726	Parylene C Oxidation (C=O stretching)	[26,29,30]
1895	Summation bands due to out-of-plane C–H wagging modes	[29,30]
2341, 2360	CO_2_ absorption	[26]
2861, 2928, 3020	C–H aliphatic stretching	[27,28,29,30]
3020, 3058	C–H aromatic stretching	[27,28]

**Table 3:** Overview of the achieved microstructure quality and observed defects by varying parameter sets of the hot embossing process.

Heating Temperature [°C]	Hot Embossing Force [kN]	Demolding Speed [mm/s]	Demolding Temperature [°C]	Structure Quality
295	25	0.2	40	Blurred edges, release-agent residues
305	25	0.2	40	Release-agent residues
315	25	0.2	40	Good
325	25	0.2	40	Good
350	25	0.2	40	Good
305	15	0.2	40	Blurred edges, incomplete structures
305	20	0.2	40	Blurred edges, incomplete structures
305	25	1.0	40	Partial delamination
305	25	0.5	40	Partial delamination
305	25	0.2	100	Partial delamination
305	25	0.2	60	Partial delamination
305	25	0.2	30	Good

**Table 4:** Experimentally determined optimal process parameters for the hot embossing of Parylene C.

Heating Temperature [°C]	Hot Embossing Force [kN]	Demolding Speed [mm/s]	Demolding Temperature [°C]	Release Agent Usage
305	25	0.2	40	Yes

**Table 5:** Overview of the shim pattern dimensions and resulting microstructures fabricated by hot embossing at 305 °C with 25 kN and demolding at 40 °C with 0.2 mm/min.

Structure Height [µm]	Structure Diameter [µm]	Aspect Ratio [a.u.]	SEM Image
10	10	1.0	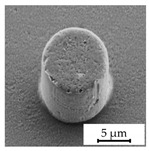
10	20	0.5	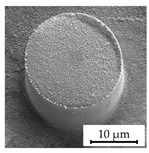
20	20	1.0	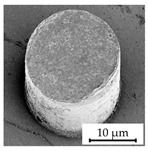
30	20	1.5	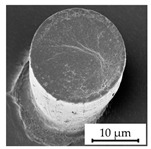

## Data Availability

The original contributions presented in the study are included in the article, further inquiries can be directed to the corresponding author.
